# Supplementary material for: The Synergistic Effect of Phosphonic and Carboxyl Acid Groups for Efficient and Stable Perovskite Solar Cells
Source: Materials (Basel). 2023 Nov 24;16(23):7306. doi: 10.3390/ma16237306 (PMC10707214; doi:10.3390/ma16237306)
Supplement: Supplementary file 1 [file materials-16-07306-s001.zip › materials-2731118-supplementary mateial.pdf]

Supporting Information

The synergistic effect of phosphonic and carboxyl acid groups for efficient and stable perovskite solar cells

Kaihuai Du[a, ‡], Aili Wang[b, ‡], Yue Li[a], Yibo Xu[a], Lvzhou Li[b, *], Ningyi Yuan[a, *], Jianning Ding[a, b, *]

Highlights

- -H_2PO_3 and -COOH play a synergistic modification role as the buried bottom interface.
- The large area uniformity of CBD SnO_2 is improved by self-assembly.
- The SnO_2 modified by mixed SAMs shows more appropriate energy levels alignment.

S1. Experimental Section

Materials. Hydrochloric acid (HCl, 37 wt.% in water) was purchased from Sinopharm. $SnCl_2 \cdot 2H_2O$ (> 99.98%) and urea (>99.0%), thioglycolic acid (TGA,≥99.0%), chlorobenzene (CB,≥99.9%), isopropanol (IPA,≥99.9%) and acetonitrile (≥99.9%) were purchased from Sigma-Aldrich. Dimethyl sulfoxide (DMSO) (≥99.9%), and dimethylformamide (DMF) (≥99.9%) were purchased from Alfa Aesar. FAI (≥99.5%), PbI_2 (99.99%), $MAPbBr_3$ (≥99.5%), MACl (≥99.5%), PEAI (≥99.5%), Spiro-OMeTAD (≥99.8%), 4-tert-Butylpyridine (tBP,≥96%), Co-TFSI salt (≥99.5%) and Li-TFSI salt (≥99.5%) were purchased from Xi'an Polymer Technology Corp. (aminomethyl)phosphonic acid (≥98%) and glycine (99%) were purchased form Adamas.

CBD SnO_2. The SnO_2 layer is deposited on the surface of cleaned FTO by chemical bath deposition (CBD). The CBD solution was prepared by mixing 5.625 g of urea, 1.2375 g of $SnCl_2 \cdot 2H_2O$, 5.625 mL of HCl, 112.5 μL of TGA, and per 450 mL of DI water. The FTO substrates and the CBD solution were loaded onto a glass reaction vessel and reacted at 94 °C in oil bath for 5.5 hours. Then, the SnO_2 deposited FTO substrates were removed from the reaction vessel and cleaned via sonication with DI water and ethanol for 10 min each.[1]

Preparation of perovskite solution. The perovskite precursor solution is prepared by

mixing 1.4 M FAI, 1.53 M PbI_2, 0.5 M MACl, 0.8 mol% $MAPbBr_3$ in 1 mL mixture solvent (volume ratio, DMF: DMSO = 8:1).

Device fabrication. The FTO/SnO_2 substrates were annealed at 170 °C for 60 minutes and treated with UV-Ozone for 20 min. Then the FTO/SnO_2 substrate was immersed in small molecule aqueous solution for 20 min, followed by drying with nitrogen, and finally placed in a 100 °C hot stage for annealing for 5 min. The prepared FTO FTO/SnO_2/SAM were transferred to the glove box. The perovskite film was spin-coated on the substrates at 1000 rpm for 10 s, and 5000 rpm for 30 s, and chlorobenzene was dropped at 20 s after start, then annealed at 100 °C for 60 min. For the 2D perovskite passivation, PEAI (15mM in IPA) was deposited at 5000 rpm for 30 s. For HTL, the 25 μL Spiro-OMeTAD solution was spin-coated on the perovskite layer at 3800 rpm for 30s. The Spiro-OMeTAD solution was prepared by dissolving 101.92 mg of Spiro-OMeTAD, 24.4 μL Li-TFSI salt (520 mg·L-1 in acetonitrile solution), 49.6 μL FK209 salt (300 mg·in 1 mL acetonitrile solution), and 45.4 μL tBP solution in 1 mL chlorobenzene. Finally, 100 nm of Au is deposited via thermal evaporation.

S2. Characterizations and measurements.

The component and work function of Perovskite (SC DMI) were measured by XPS and UPS (Thermo Scientific EscaLab 250Xi). FTIR spectra was performed using FTIR-650S (GANGDONG, China). The thin film samples and the liquid samples were both analyzed on KBr substrates. X-ray diffraction (XRD) patterns were analysis with Cu Kα radiation (D-max 2500 PC, Rigaku Corporation, Japan). The UV-Vis spectra of perovskite films were measured using the ultraviolet-visible spectrophotometer (CARY 100 UV-Vis, Agilent technologies, USA). PL (excitation at 520 nm) and TRPL characteristics were obtained using FLS980 (Edinburgh Instruments Ltd.). The morphology and crystallinity of the perovskite films were studied with field-emission scanning electron microscopy (FE-SEM; SUPRA, Zeiss, Germany). The *J–V* measurements were determined via a Keithley 2400 source and the solar simulator with standard AM 1.5 G (Newport Oriel 94043A, USA, AM1.5, 100 mW·cm^{-2}). PL mapping was achieved with the excitation light source of a continuous wave 375 nm 200 KHz

laser. The scanning step length in X and Y directions of ×10 objective lens is 20 μm, and the scanning step length in X and Y directions of ×100 objective lens is 1 μm.

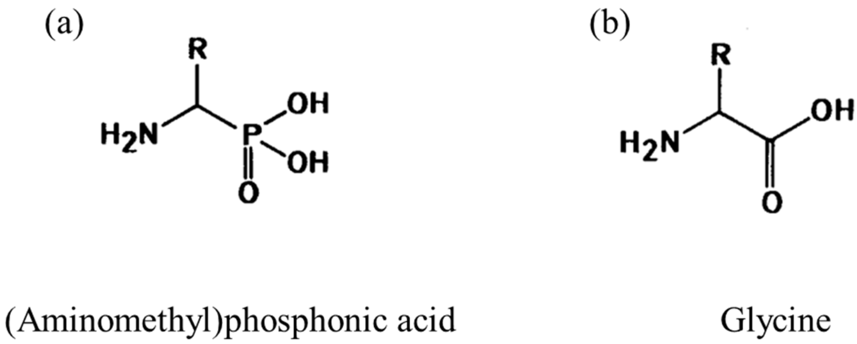

Figure S1. Structural formula of a) P-SAM and b) C-SAM. (-R, $-CH_2$)

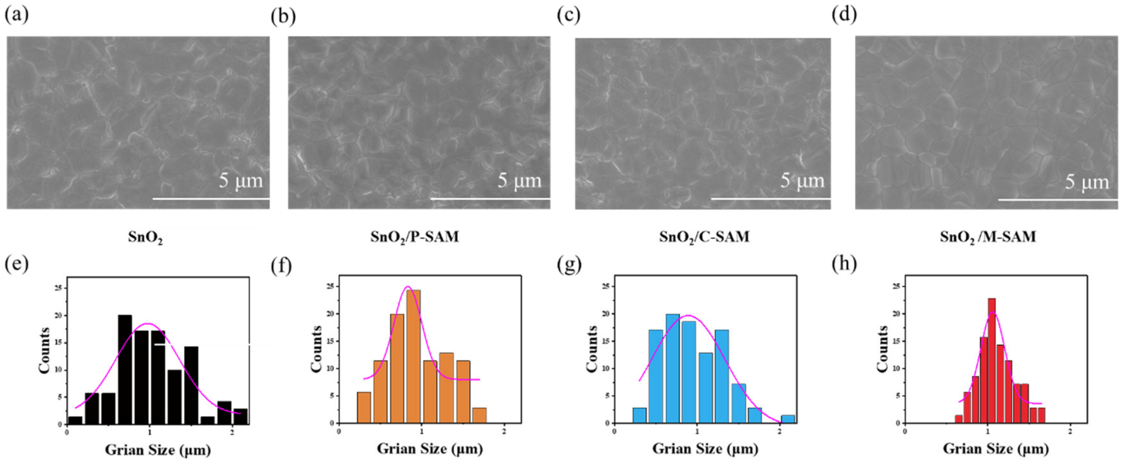

Figure S2. SEM images of perovskite surface based on a) SnO_2, b) SnO_2/P-SAM SnO_2, c) SnO_2/C-SAM SnO_2 and d) SnO_2/M-SAM. The perovskite grain size of e) SnO_2, f) SnO_2/P-SAM SnO_2, g) SnO_2/C-SAM SnO_2 and h) SnO_2/M-SAM.

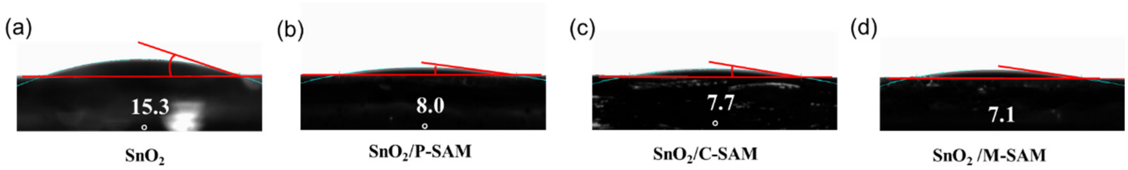

Figure S3. Contact angles of a) SnO_2, b) SnO_2/P-SAM, c) SnO_2/C-SAM, and d) SnO_2/M-SAM as substrates.

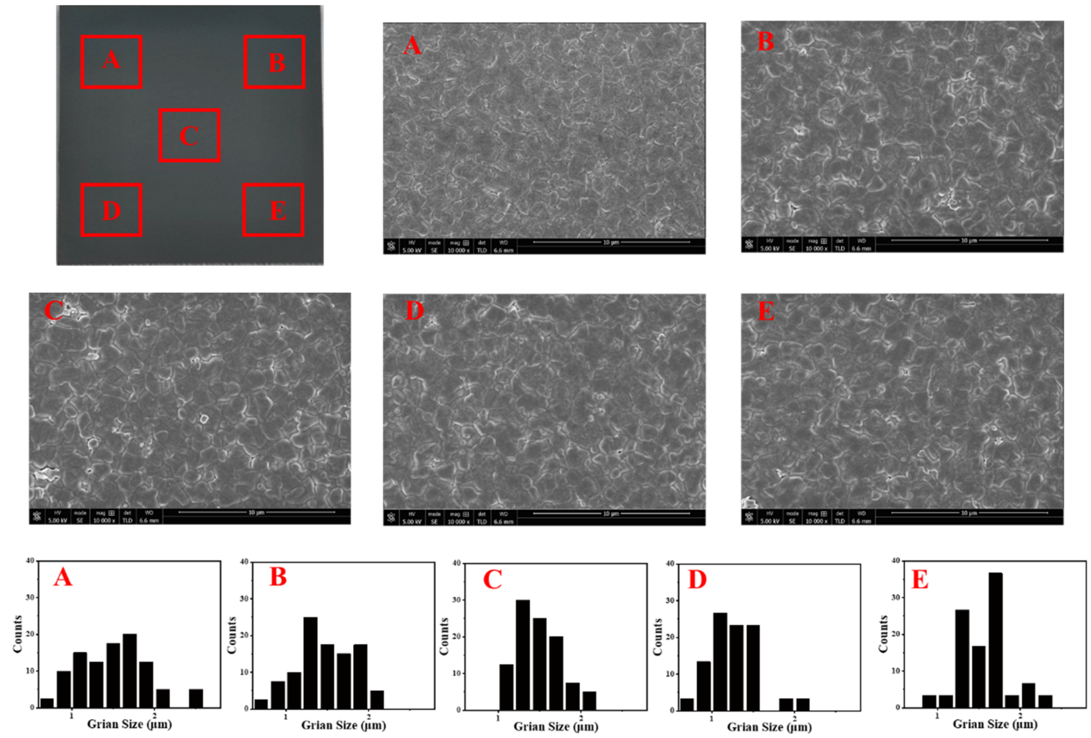

Figure S4. SEM images for five different locations of the 4×4 cm^2 perovskite film deposited on SnO_2 substrate. The scale bar is 10 μm. The distribution of the grain size is plotted in histogram for comparison.

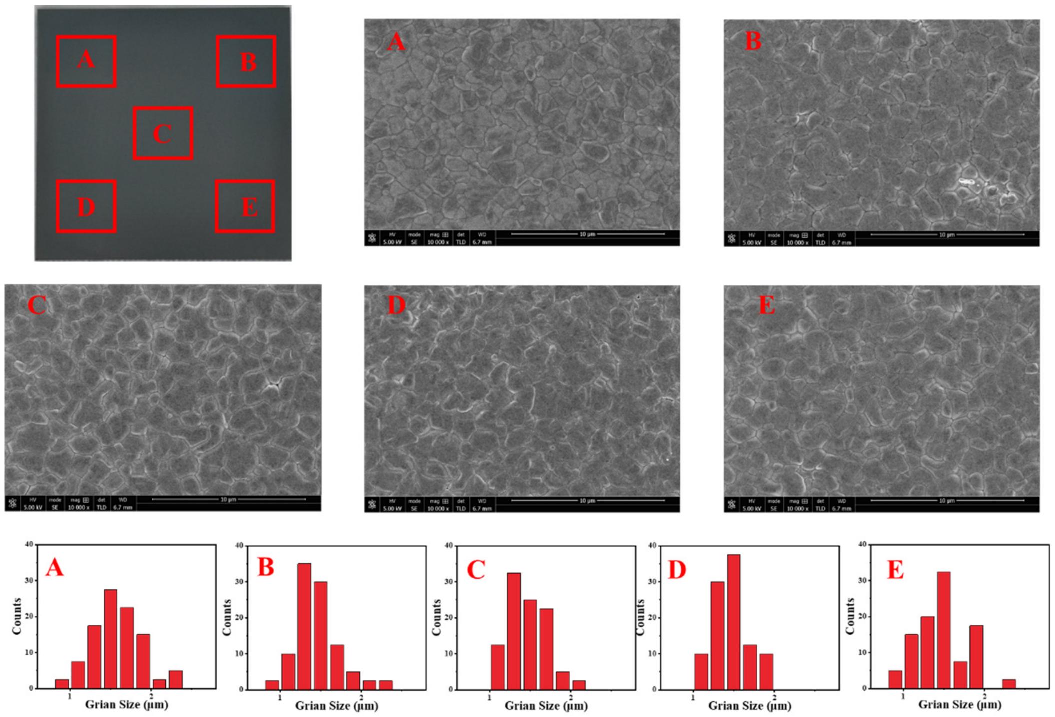

Figure S5. SEM images for five different locations of the 4×4 cm^2 perovskite film deposited on SnO_2/M-SAM substrate. The scale bar is 10 μm. The distribution of the grain size is plotted in histogram for comparison.

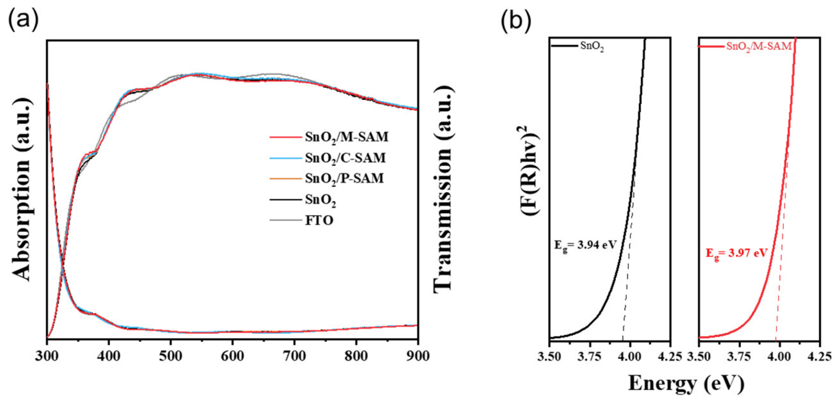

Figure S6. a) Transmission and absorption spectra FTO, SnO_2 SnO_2/P-SAM, SnO_2/C-SAM and SnO_2/M-SAM films. b) Tauc plots of pristine SnO_2 and SnO_2/M-SAM films.

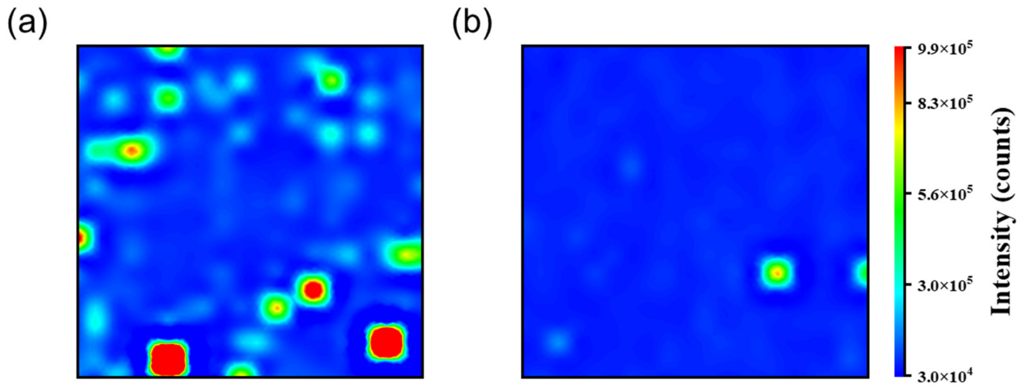

Figure S7. PL intensity imaging of SnO_2/perovskite and SnO_2/M-SAM/perovskite films (size of 0.02×0.02 mm^2).

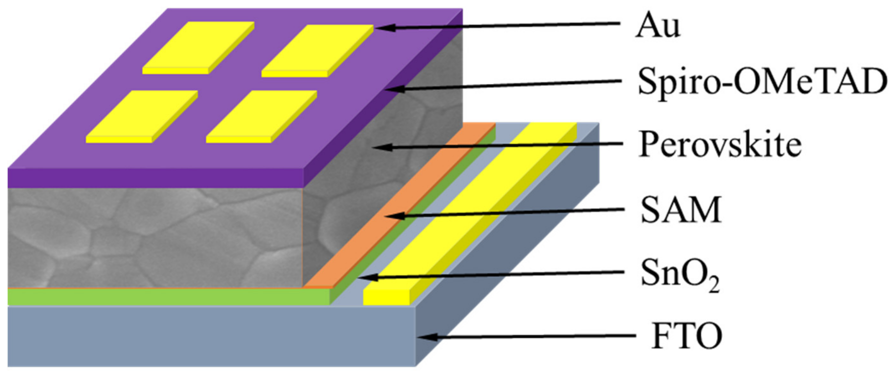

Figure S8. PSCs with the structure of FTO/SnO_2/SAM/Perovskite/Spiro-OMeTAD/Au.

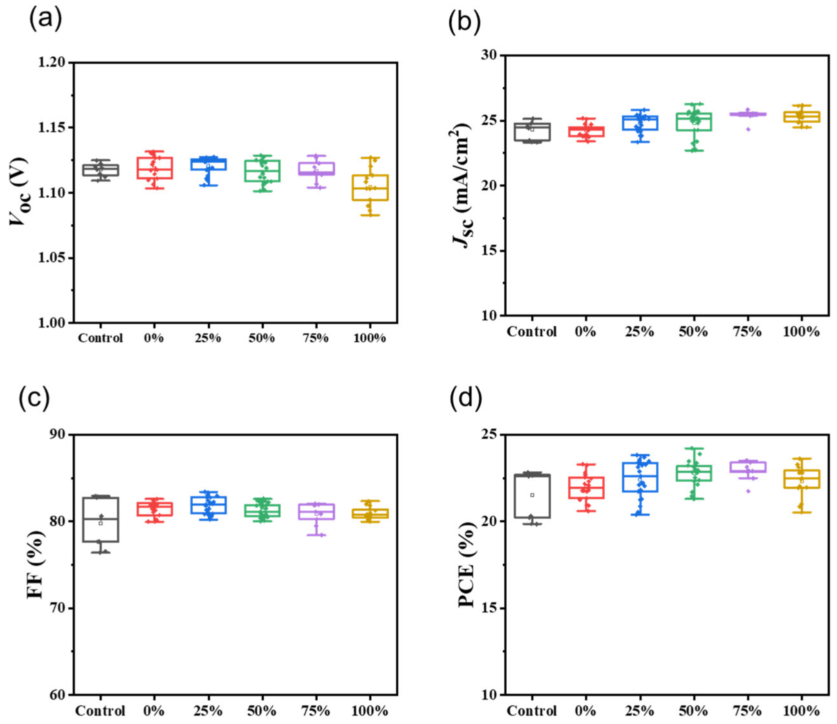

Figure S9. The PSCs performance a) V_{OC}, b) J_{SC}, c) FF and d) PCE of the devices with different molar ratios of P-SAM and C-SAM.

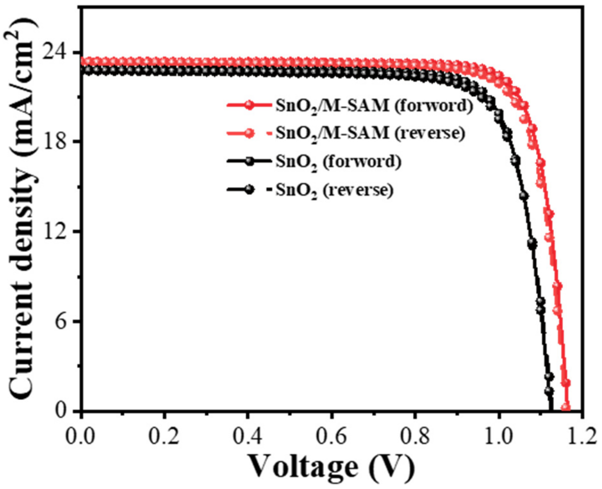

Figure S10. *J-V* curves for control and M-SAM modified PSCs under reverse and forward scans (at illumination of 100 mW/cm^2).

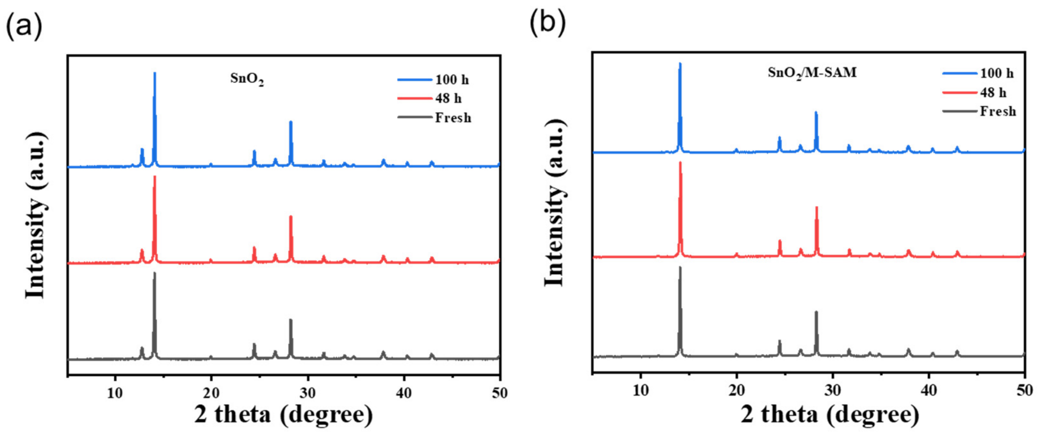

Figure S11. XRD patterns for a) SnO_2/perovskite and b) SnO_2/M-SAM/perovskite films aged at a RH≈40%, T: ≈25 °C.

Table S1. Summarized parameters of energy band structure for SnO_2, SnO_2/M-SAM (on FTO substrates).

	E_g (eV)	E_{cutoff} (eV)	$E_{fermi\ edge}$ (eV)	E_V (eV)	E_F (eV)	E_C (eV)
SnO_2/M-SAM	3.97	16.19	4.58	9.61	5.03	5.64
SnO_2	3.94	15.41	4.27	10.08	5.81	6.14

Table S2. Fitted parameters of the TRPL curves for SnO_2/perovskite SnO_2/P-SAM/perovskite, SnO_2/C-SAM/perovskite and SnO_2/M-SAM/perovskite films (on glass substrates).

	τ_1 (μs)	A_1	τ_2 (μs)	A_2	τ_{ave} (μs)
SnO_2/M-SAM	0.013	1.81	1.090	0.64	1.056
SnO_2/C-SAM	0.013	2.64	1.191	0.69	1.142
SnO_2/P-SAM	0.020	0.83	1.315	0.76	1.294
SnO_2	0.020	0.87	1.499	1.03	1.482

Table S3. Fitted parameters of the TRPL curves for glass/perovskite, glass/P-SAM/perovskite, glass/C-SAM/perovskite and glass/M-SAM/perovskite films.

	τ_1 (μs)	A_1	τ_2 (μs)	A_2	τ_{ave} (μs)
Glass/M-SAM	0.037	0.28	1.204	0.85	1.192
Glass/C-SAM	0.031	0.45	0.935	0.69	0.916
Glass/P-SAM	0.026	0.84	0.646	0.64	0.614
Glass	0.019	0.83	0.581	0.58	0.556

Table S4. Fitted parameters of the PL intensity imaging for SnO_2/perovskite SnO_2/P-SAM/perovskite, SnO_2/C-SAM/perovskite and SnO_2/M-SAM/perovskite films (on glass substrates).

	τ_1 (μs)	A_1	τ_2 (μs)	A_2	τ_3 (μs)	A_3	τ_{ave} (μs)
SnO_2/M-SAM	0.046	0.38	0.408	0.33	3.77	0.29	0.107
SnO_2/C-SAM	0.046	0.34	0.364	0.44	2.55	0.21	0.114
SnO_2/P-SAM	0.016	0	0.089	0.59	0.596	0.41	0.130
SnO_2	0.096	0.29	0.177	0.53	0.533	0.15	0.156

Table S5. Fitted parameters of the PL intensity imaging for SnO_2/perovskite and SnO_2/M-SAM/perovskite films (on glass substrates).

	τ_1 (μs)	A_1	τ_2 (μs)	A_2	τ_3 (μs)	A_3	τ_{ave} (μs)
SnO_2/M-SAM	0.024	0.33	0.217	0.50	2.75	0.17	0.062
SnO_2	0.046	0.46	0.312	0.43	5.77	0.11	0.086

Table S6. Photovoltaic parameters for SnO_2 and M-SAM modified PSCs under reverse scan and forward scan (HI = ($PCE_{forward}$-$PCE_{reverse}$)/$PCE_{forward}$).

	V_{OC} (V)	J_{SC} (mA/cm^2)	FF (%)	PCE (%)	HI
SnO_2/M-SAM forward	1.16	25.54	82.45	22.45	0.024
SnO_2/M-SAM reverse	1.16	24.17	80.87	21.91	
SnO_2 forward	1.12	22.80	79.99	20.42	0.025
SnO_2 reverse	1.12	22.70	78.32	19.91	

Table S7. Calculated parameters and trap densities (N_{trap}) of perovskite films grown on SnO_2 and SnO_2/M-SAM substrates for electron only devices.

	L (nm)	ε	Area (cm^2)	V_{TFL} (V)	N_{trap} (cm^{-3})
SnO_2/M-SAM	480	62	0.09	0.96	1.94×10^{16}
SnO_2	480	62	0.09	1.10	2.22×10^{16}

Table S8. The modification of CBD SnO_2 for getting the improved performance of PSCs are listed below.

Surface passivation	V_{OC} (V)	J_{SC} (mA/cm2)	FF (%)	PCE (%)
Mercaptosuccinic acid [2]	1.14	22.95	80.9	21.16
NaBF4 [3]	1.10	23.51	80.48	20.82
Periodic acid [4]	1.09	25.02	81.55	22.25
This Work	1.16	25.54	83.07	24.69

References and Notes

1. Yoo, J. J.; Seo, G.; Chua, M. R.; Park, T. G.; Lu, Y.; Rotermund, F.; Kim, Y.-K.; Moon, C. S.; Jeon, N. J.; Correa-Baena, J.-P.; Bulovic, V.; Shin, S. S.; Bawendi, M. G.; Seo, J., Efficient perovskite solar cells via improved carrier management. *Nature* **2021,** 590, (7847).
2. Zhang, J. B.; Bai, C.; Dong, Y.; Shen, W. J.; Zhang, Q.; Huang, F. Z.; Cheng, Y. B.; Zhong, J., Batch chemical bath deposition of large-area SnO₂ film with mercaptosuccinic acid decoration for homogenized and efficient perovskite solar cells. *Chemical Engineering Journal* **2021,** 425, 8.
3. Soe, K. T.; Thansamai, S.; Thongprong, N.; Ruengsrisang, W.; Muhammad, I. A.; Ketsombun, E.; Supruangnet, R.; Kaewprajak, A.; Kumnorkaew, P.; Saetang, V.; Supasai, T.; Rujisamphan, N., Simultaneous Surface Modification and Defect Passivation on Tin Oxide-Perovskite Interfaces using Pseudohalide Salt of Sodium Tetrafluoroborate. *Solar Rrl* **2023,** 7, (1), 15.
4. Wu, Z. Y.; Su, J. Z.; Chai, N. Y.; Cheng, S. Y.; Wang, X. Y.; Zhang, Z. L.; Liu, X. L.; Zhong, H.; Yang, J. F.; Wang, Z. P.; Liu, J. B.; Li, X.; Lin, H., Periodic Acid Modification of Chemical-Bath Deposited SnO₂ Electron Transport Layers for Perovskite Solar Cells and Mini Modules. *Advanced Science* **2023,** 10, (20), 9.
